# Phylogenomics identifies parents of naturally occurring tetraploid bananas

**DOI:** 10.1186/s40529-024-00429-9

**Published:** 2024-07-12

**Authors:** Yu-En Lin, Hui-Lung Chiu, Chung-Shien Wu, Shu-Miaw Chaw

**Affiliations:** 1 Department of Biochemical Science and Technology, National Taiwan University, Taipei, 106319 Taiwan; 2 Biodiversity Research Center, Academia Sinica, Taipei, 11529 Taiwan; 3 Crop Genetic Resources and Biotechnology Division, Taiwan Agricultural Research Institute, Taichung, 413008 Taiwan

**Keywords:** Banana, Hybridization, Polyploidization, Apomeiosis, Triploid bridge

## Abstract

**Background:**

Triploid bananas are almost sterile. However, we succeeded in harvesting seeds from two edible triploid banana individuals (Genotype: ABB) in our conservation repository where various wild diploid bananas were also grown. The resulting rare offspring survived to seedling stages. DNA content analyses reveal that they are tetraploid. Since bananas contain maternally inherited plastids and paternally inherited mitochondria, we sequenced and assembled plastomes and mitogenomes of these seedlings to trace their hybridization history.

**Results:**

The coding sequences of both organellar genomic scaffolds were extracted, aligned, and concatenated for constructing phylogenetic trees. Our results suggest that these tetraploid seedlings be derived from hybridization between edible triploid bananas and wild diploid *Musa balbisiana* (BB) individuals. We propose that generating female triploid gametes via apomeiosis may allow the triploid maternal bananas to produce viable seeds.

**Conclusions:**

Our study suggests a practical avenue towards expanding genetic recombination and increasing genetic diversity of banana breeding programs*.* Further cellular studies are needed to understand the fusion and developmental processes that lead to formation of hybrid embryos in banana reproduction, polyploidization, and evolution.

**Supplementary Information:**

The online version contains supplementary material available at 10.1186/s40529-024-00429-9.

## Background

Musaceae, the banana family, comprises approximately 91 species generally assigned to three genera, *Ensete*, *Musella*, and *Musa*, although the taxonomic status of *Musa* has been controversial (Christenhusz and Byng 2016). To date, four *Musa* sections are recognized: *Australimusa* (2*x* = 20), *Callimusa* (2*n* = 2*x* = 18 or 20), *Eumusa* (2*n* = *2x* = 22), and *Rhodochlamys* (2*n* = 2*x* = 22). Most edible bananas belong to *Eumusa* (Cheesman 1947; Debnath et al. 2019).

An estimated 137 million tons of bananas were produced in 2021, with export values exceeding 12.7 billion US dollars (FAOSTAT Statistical Database 2023). Profits from the banana industry are particularly important to low-income nations. Unfortunately, cultivated bananas are susceptible to numerous diseases, especially *Fusarium oxysporum* f. sp. *cubense* tropical race 4 (Foc TR4) (Ploetz 2015). Developing resistant cultivars through hybridization is an important strategy for banana disease control (Siamak and Zheng 2018).

Banana cultivars are mainly derived from intra- or inter-specific hybridizations between *M. acuminata* (A genome or AA genotype) and *M. balbisiana* (B genome or BB genotype) (Cheesman 1947; Simmonds and Shepherd 1955). These crosses result in varieties/cultivars that can be diploid (2*n* = 2*x* = 22), triploid (2*n* = 3*x* = 33), and very rarely tetraploid (2*n* = 4*x* = 44). A common strategy for introducing disease resistance is to develop tetraploids (4*x*) from 3*x* × 2*x* crosses. Triploid varieties can produce unreduced female gametes that can develop into embryos once fertilized. Banana varieties from the Gros-Michel (AAA), Mysore (AAB), Pome/Prata (AAB), Plantain (AAB), and Pisang Awak (ABB) are examples of these (Bakry and Horry 1992). Many breeders have developed new disease-resistant bananas using this scheme (Jenny et al. 2003).

In nature, most polyploidization events involve a two-step reduced-unreduced gamete fusion mechanism called the “triploid bridge” (Ramsey and Schemske 1998). This mechanism requires the production of tetraploids via triploid intermediates. First, a regular haploid (*n*, reduced) gamete fuses to a rare diploid (2*n*, unreduced) gamete to form a triploid individual, called a neo-triploid. This neo-triploid plant then serves as a “bridge” that provides unreduced euploid (3*n*) gametes and thus can cross with reduced (*n*) gametes of diploid individuals to generate tetraploid offsprings (Ramsey and Schemske 1998; De Storme and Geelen 2013; Hojsgaard 2018). The efficiency of triploid bridges depends on the successful production of unreduced euploid gametes from triploid parents (Köhler et al. 2010; Wang et al. 2016; Hojsgaard 2018). Ramsey and Schemske (1998) observed that euploid gametes appear to be more frequent in nature than expected. To further interpret this phenomenon, Hojsgaard (2018) proposed that apomixis, characterized by the transient production of apomeiotic gametes, provides unreduced gametes and may increase the likelihood of triploid bridges.

Organelles are differentially inherited in bananas: plastids are maternally inherited, while mitochondria come from fathers (Fauré et al. 1994). This unusual cytoplasmic inheritance provides a helpful and convenient avenue for tracing hybrid banana origins (Carreel et al. 2002; Boonruangrod et al. 2008; Wu et al. 2021). Over the past five years, we collected seeds from two edible triploid bananas (ABB) that grew next to various wild diploid bananas in the *Musa* germplasm repository at Taiwan Agricultural Research Institute (TARI). Two of the collected seeds have germinated into seedlings.

This study aims to determine the ploidy of the seedlings germinated from the seeds of edible triploid ABB bananas and to clarify their parentage. The mechanism underlying tetraploid banana generation is also discussed.

## Materials and methods

### Sampling of *Musa* taxa

**Fig. 1:**
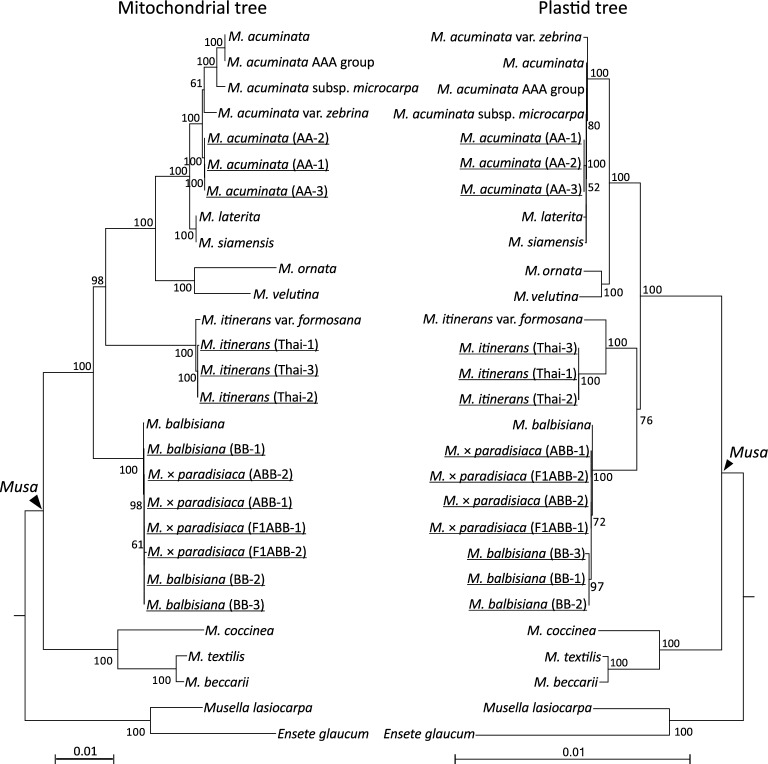
Maximum likelihood phylogenomic trees based on concatenations of mitochondrial (left-hand side) and plastid (right-hand side) genes. Taxa sequenced in this study are underlined. Bootstrap values are shown along branches. Trees are condensed under a 50% majority rule

**Fig. 2:**
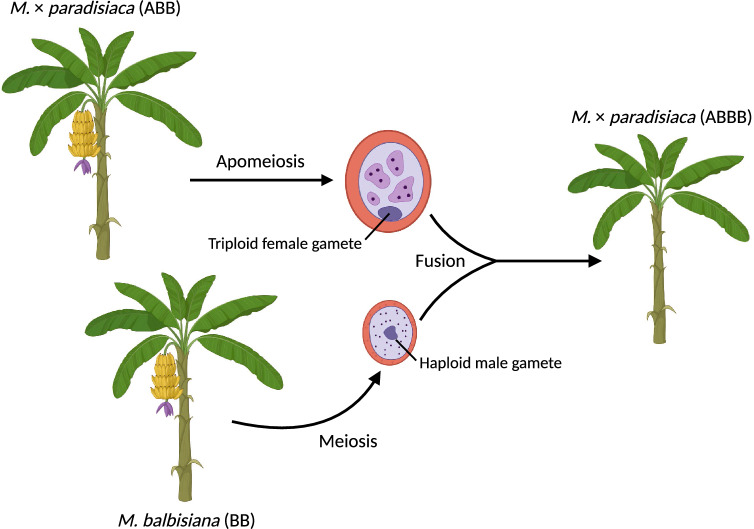
A scheme showing formation of tetraploid bananas via fusion of female triploid and male haploid gametes

We collected 13 samples: three *M. acuminata* individuals (designated as AA-1, AA-2, and AA-3 because of their AA genotype), three *M. balbisiana* individuals (BB-1, BB-2, and BB-3 because of their BB genotype), three *M. itinerans* Thailand variants (Thai-1, Thai-2, and Thai-3), two triploid bananas of *M.* × *paradisiaca* (ABB-1 and ABB-2), and two seedlings of *M.* × *paradisiaca* (F1ABB-1 and F1ABB-2) germinated from the seeds of the ABB-1 and ABB-2 triploid bananas. We sampled three *M. itinerans* individuals because they grew next to the triploid bananas and successful hybridization between *M. itinerans* var. *formosana* and *M. balbisiana* was reported before (Chiu et al. 2017). These 13 sampled individuals are growing well in the experimental farm of *Musa* germplasm repository at TARI in Taichung city. Their specimens are deposited in Biodiversity Research Museum, Academia Sinica (voucher numbers: Chaw1610–1622).

### Plastome and mitogenome assembly and annotation

Two grams of flash leaves from each sampled individual were collected for DNA extraction using a modified CTAB method (Stewart and Via 1993) with 0.1% polyvinylpyrrolidone (PVP-40, Sigma). DNA libraries were constructed and then sequenced on an Illumina NovaSeq 6000 platform in Genomics Biotech Company (New Taipei City, Taiwan) to generate pair-end reads of 2 × 150 bp. We used Trimmomatic-0.39 (Bolger et al. 2014) to remove adapters and low-quality bases with the parameters of “ILLUMINACLIP: TruSeq3-PE.fa:2:30:10 LEADING: 3 TRAILING: 3 SLIDINGWINDOW: 4:15 MINLEN: 36”. We used Getorganelle v1.7.7 (Jin et al. 2020) to assemble plastomes, and annotated the genomes using Plastid Genome Annotator (PGA) (Qu et al. 2019). Mitogenomic scaffolds were obtained using SPAdes 3.12 (Bankevich et al. 2012), followed by annotations using Geneious Prime 2023.2 (https://www.geneious.com/) based on a self-built database and manual correction.

### Flow cytometry analysis

Fresh banana leaves were chopped with 250 µL isolation buffer (200 mM Tris, 4 mM MgCl2-6H2O, and 0.5% Triton X-100) and mixed well. The mixture was filtered through a 40-μm nylon mesh. The filtered suspensions were incubated with DNA fluorochrome (50 μg/mL propidium iodide containing RNase A) for 30 min at 37 °C. The samples were analyzed using a CytoFLEX S Flow Cytometer (Beckman Coulter Life Science) in the Institute of Plant and Microbial Biology, Academia Sinica. For each examined banana, three independent replicates of analyses were conducted using *M. acuminata* (AA) as the reference.

### Construction of mitochondrial and plastid trees

IQtree2 (Minh et al. 2020) was used to construct mitochondrial and plastid phylogenetic trees based on the concatenation of 42 mitochondrial (3 rRNAs and 39 protein-coding genes) and 79 plastid protein-coding genes, respectively. We set the “MFP” option that allowed IQtree2 to automatically evaluate the best-fit substitutional model according to the Bayesian information criterion. As a result, the best-fit models were TVM + F + R2 and TVM + F + R4 for constructing the mitochondrial and plastid trees, respectively. Branch support was assessed using 1000 non-parametric bootstrap replicates. Trees were visualized in Mega 7 (Kumar et al. 2016).

## Results and discussion

### Ploidy determination using flow cytometry

As expected, our flow cytometry analysis indicates that diploids (AA) contain less DNA than triploids (ABB). Using the haploid genome (1C = 523 Mb) of the diploid banana (D'Hont et al. 2012) as the reference, we estimated the examined triploid banana’s genome to be 636.7 ± 15.7 Mb. In contrast, the F1ABB banana’s genome was estimated to be 1098 ± 15.7 Mb, approximately double of the diploid (Figure S1). Therefore, the F1ABB banana is tetraploid derived from a triploid maternal parent. This result also affirms that a successful syngamy has taken place in a triploid ABB banana.

### Using mitochondrial and plastid trees to trace parentage

Fauré et al. (1994) discovered that while mitochondria are paternally inherited in bananas, plastids come from the maternal parent. This facilitates tracing hybrid parentage in banana breeding programs (Carreel et al. 2002; Boonruangrod et al. 2008; Wu et al. 2021).

Our two tetraploid bananas, their triploid maternal parents, and all sampled *M. balbisiana* accessions constitute a strongly supported clade in the mitochondrial tree (Fig. 1; BS = 100%). This clade is also observed in our plastid tree (Fig. 1; BS = 100%). Therefore, the two organelle trees strongly suggest that (1) the paternal origin of the two tetraploid bananas (F1ABB-1 and F1ABB-2) is *M. balbisiana*, and (2) the tetraploid bananas’ plastids come from their triploid mothers, whose plastids were previously transmitted from *M. balbisiana*. This result also further supports the idea that the genotype of the two tetraploids is ABBB.

### Apomeiosis-associated female triploid gametes interpret formation of tetraploid bananas

Our flow cytometry analysis and phylogenomic results suggest that a successful syngamy occurred between maternal triploid ABB and paternal diploid *M. balbisiana*. Hojsgaard (2018) proposed that triploid bridges are the chief contributors to polyploidization in the wild. If this is true, the triploid AAB banana has generated the euploid female gametes that went on to form the tetraploid seeds we have investigated here.

Figure 2 illustrates the hypothetical mechanism underlying tetraploid banana formation via fusion of female triploid and male haploid gametes. Initially, we planted two triploid ABB banana cultivars in the TARI experimental farm where several diploid bananas also grew. It was previously noted that the combination of divergent and unbalanced genome sets facilitates genomic shock leading to apomeiosis and generation of unreduced gametes (Comai et al. 2000; Adams and Wendel 2005; Talent and Dickinson 2007; Madlung and Wendel 2013; Hojsgaard 2018). Furthermore, the high fruiting rate of triploid bananas likely also contributes to the frequent occurrence of apomeiosis. However, this mechanism is not impossible in the case presented here because the two mother triploid bananas produced unreduced female gametes (2*n* = 3x = 33). In addition, we ruled out the self-crossing as bananas are not self-pollinating plants. In our case, apomeiosis allowed the triploid ABB bananas to develop euploid female gametes, which subsequently fused with sperm released from haploid male pollen of the nearby diploid *M. balbisiana*. Together, they ultimately produced seeds that germinated as tetraploid ABBB bananas (Fig. 2). This also demonstrates a feasible way to facilitate genetic variation and polyploidization in banana breeding programs.

## Conclusion

The triploid ABB bananas studied here are a variety of Kluai Namwa bananas, commonly cultivated in Taiwan. Our molecular evidence demonstrates that F1ABB is a tetraploid hybrid of Kluai Namwa banana (ABB) and *M. balbisiana* (BB)*.* We propose that the apomeiosis-associated triploid bridge plays a key role in breeding of polyploid bananas, as exemplified by our viable tetraploid seedlings. Specifically, our data not only clarify the parental origin of the seeds produced from triploid bananas but also present a feasible case for breeding of tetraploid banana hybrids via the female triploid bridge. Therefore, growing triploid and diploid accessions together may expedite the discovery of new germplasm that can increase genetic resources for the banana industry*.* Further cellular studies on the fusion and developmental processes leading to hybrid embryos are needed to deepen our knowledge of banana reproduction, polyploidization, and evolution.

### Supplementary Information

Supplementary Material 1. Figure S1. Flow cytometry analyses revealing the relative DNA content (x-axis) of diploid (AA), triploid (ABB), and tetraploid (F1ABB) bananas. The estimated genome size (1C) is shown on the right panel.

## Data Availability

All DNAseq reads used for the genome assembly are available in the NCBI SRA database under a BioProject number: PRJNA1066417. The assembled organellar genomes are deposited in the GenBank (Plastomes: LC792611‒LC792623; mitochondrial scaffolds: LC794909‒LC795246).
