# Supplementary material for: Phylogenomics identifies parents of naturally occurring tetraploid bananas
Source: Bot Stud. 2024 Jul 12;65:19. doi: 10.1186/s40529-024-00429-9 (PMC11245450; doi:10.1186/s40529-024-00429-9)
Supplement: Supplementary file 1 — Supplementary Material 1. Figure S1. Flow cytometry analyses revealing the relative DNA content (x-axis) of diploid (AA), triploid (ABB), and tetraploid (F1ABB) bananas. The estimated genome size (1C) is shown on the right panel. [file 40529_2024_429_MOESM1_ESM.pdf]

Figure S1

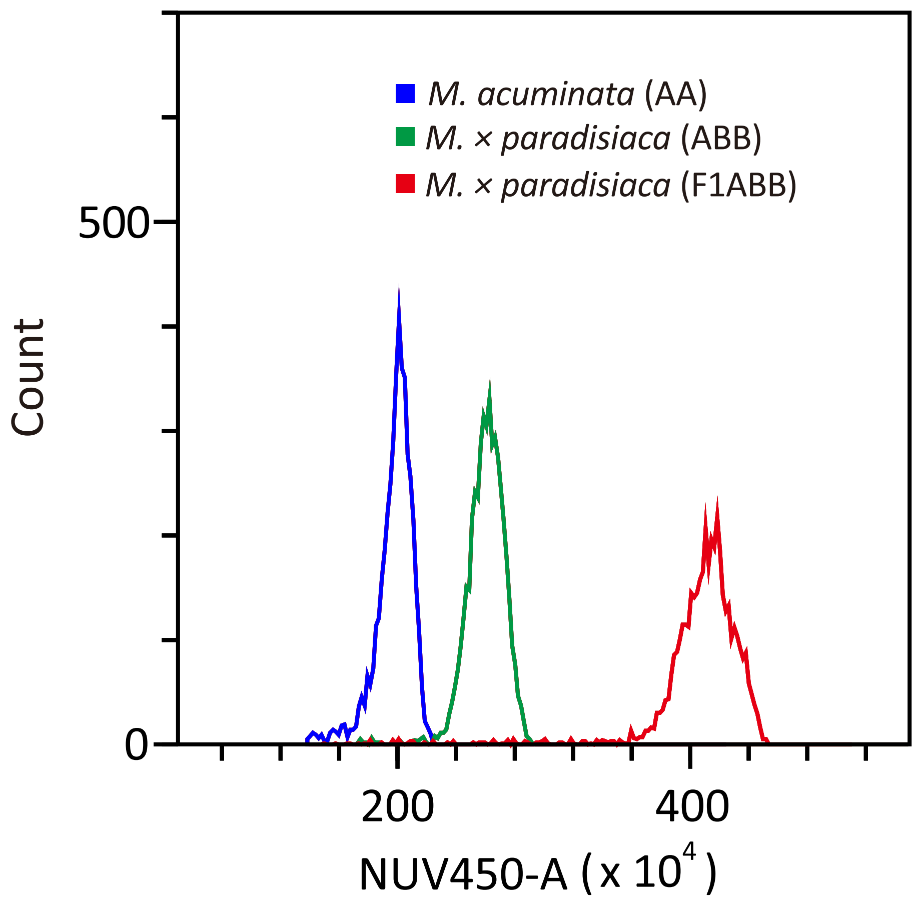

Estimated genome size (1C)

M. acuminata (reference): 523 Mb

M. × *paradisiaca* (ABB): 636.7 ± 15.7 Mb

M. × *paradisiaca* (F1ABB): 1,098 ± 3.8 Mb
